# Comparative Pain and Constitutional Affections from Couching and Extracting the Cataract

**Published:** 1835

**Authors:** 


### III.—Comparative Pain and Constitutional Affections from Conching and Extracting the Cataract.

The people, and many practitioners, seem to have an opinion, that conching is attended with less pain and constitutional disturbance, than extraction; but from considerable experience we are convinced that this is not the fact. The introduction of the needle through the sclerolitæ is generally attended with greater suffering at the moment, and a much deeper state of nervous irritation, with nausea, than the incision of the corneo; and the pressure of the dislocated lens is more severely felt by the patient, than its escape from the eye. These facts, with many others, lead us to prefer extraction to depression, provided, of course, that the state of the cornea and anterior chamber be such as to favor the former operation.

